# 21813 Changes in Electrophysiologic Activity in the Rat Visual Cortex following Traumatic Brain Injury (TBI)

**DOI:** 10.1017/cts.2021.425

**Published:** 2021-03-30

**Authors:** James Germi, Oceane Fruchet, John Wolf, Isaac Chen

**Affiliations:** Perelman School of Medicine

## Abstract

ABSTRACT IMPACT: This research aims to identify changes in visual network function after TBI as a way to define potential therapeutic targets for neuromodulation or neural tissue substrates. OBJECTIVES/GOALS: The objectives of this study are to compare neural activity in the visual cortex following TBI with cortical activity in the uninjured brain. This study aims to characterize functional changes in single neuron activity, spike-field relationships and oscillatory activity. METHODS/STUDY POPULATION: The effects of TBI will be studied by comparing electrophysiologic recordings from Long-Evans rats with a fluid percussion injury (FPI) to rats with a sham injury. Four days after the injury or sham procedure, a laminar probe with multiple electrode contacts will be chronically implanted in the ipsilesional primary visual cortex (V1). Afterwards, rats will be anesthetized weekly for 3 weeks (up to 4 weeks post-injury) to assess visual processing in response to drifting grating visual stimulation. To assess behavioral correlates, neural activity will also be recorded while rats perform a visual discrimination task in an operant, touchscreen chamber twice weekly. Recordings will be analyzed for visually evoked units, unit entrainment to local field potentials (LFPs) and evoked oscillatory activity. RESULTS/ANTICIPATED RESULTS: Consistent with other studies, our preliminary evidence from V1 recordings in naive rats has shown that individual neurons are responsive to visual stimuli, visual stimuli are associated with evoked oscillations and unit activity is correlated with LFPs. While activity of individual V1 neurons in injured animals is expected to recover to resemble activity in uninjured animals over time, patterns of functional organization in the two groups are expected to diverge over time. We anticipate that TBI-associated axonal damage, neuronal loss and changes in synaptic weights will lead to disruptions in the timing of neural activity in V1. These perturbations of neural communication within the visual system are expected to be associated with behavioral deficits in the awake, visual discrimination task. DISCUSSION/SIGNIFICANCE OF FINDINGS: This study helps define how cortical network disruption after TBI. These changes are potential targets for novel TBI therapeutics, including neuromodulation and neural tissue transplantation. Thus, this work lays the groundwork for future studies aimed at mitigating the effects of TBI with rationally designed experimental therapeutics.

